# A Technological-Based Platform for Risk Assessment, Detection, and Prevention of Falls Among Home-Dwelling Older Adults: Protocol for a Quasi-Experimental Study

**DOI:** 10.2196/25781

**Published:** 2021-08-12

**Authors:** Fátima Araújo, Maria Nilza Nogueira, Joana Silva, Sílvia Rego

**Affiliations:** 1 Escola Superior de Enfermagem do Porto (ESEP) Inovação e Desenvolvimento em Enfermagem Centro de Investigação em Tecnologias e Serviços de Saúde Porto Portugal; 2 Fraunhofer Portugal Research Center for Assistive Information and Communication Solutions Porto Portugal

**Keywords:** fall prevention, technological platform, elderly, Otago Exercise Program

## Abstract

**Background:**

According to the United Nations, it is estimated that by 2050, the number of people aged 80 years and older will have increased by 3 times. Increased longevity is often accompanied by structural and functional changes that occur throughout an individual’s lifespan. These changes are often aggravated by chronic comorbidities, adopted behaviors or lifestyles, and environmental exposure, among other factors. Some of the related outcomes are loss of muscle strength, decreased balance control, and mobility impairments, which are strongly associated with the occurrence of falls in the elderly. Despite the continued undervaluation of the importance of knowledge on fall prevention among the elderly population by primary care health professionals, several evidence-based (single or multifaceted) fall prevention programs such as the Otago Exercise Program (OEP) have demonstrated a significant reduction in the risk of falls and fall-related injuries in the elderly within community settings. Recent studies have strived to integrate technology into physical exercise programs, which is effective for adherence and overcoming barriers to exercise, as well as improving physical functioning.

**Objective:**

This study aims to assess the impact of the OEP on the functionality of home-dwelling elderly using a common technological platform. Particularly, the impact on muscle strength, balance, mobility, risk of falling, the perception of fear of falling, and the perception of the elderly regarding the ease of use of technology are being examined in this study.

**Methods:**

A quasi-experimental study (before and after; single group) will be conducted with male and female participants aged 65 years or older living at home in the district of Porto. Participants will be recruited through the network COLABORAR, with a minimum of 30 participants meeting the study inclusion and exclusion criteria. All participants will sign informed consent forms. The data collection instrument consists of sociodemographic and clinical variables (self-reported), functional evaluation variables, and environmental risk variables. The data collection tool integrates primary and secondary outcome variables. The primary outcome is gait (timed-up and go test; normal step). The secondary outcome variables are lower limb strength and muscle resistance (30-second chair stand test), balance (4-stage balance test), frequency of falls, functional capacity (Lawton and Brody - Portuguese version), fear of falling (Falls Efficacy Scale International - Portuguese version), usability of the technology (System Usability Scale - Portuguese version), and environmental risk variables (home fall prevention checklist for older adults). Technological solutions, such as the FallSensing Home application and Kallisto wearable device, will be used, which will allow the detection and prevention of falls. The intervention is characterized by conducting the OEP through a common technological platform 3 times a week for 8 weeks. Throughout these weeks, the participants will be followed up in person or by telephone contact by the rehabilitation nurse. Considering the COVID-19 outbreak, all guidelines from the National Health Service will be followed. The project was funded by InnoStars, in collaboration with the Local EIT Health Regional Innovation Scheme Hub of the University of Porto.

**Results:**

This study was approved on October 9, 2020 by the Ethics Committee of Escola Superior de Enfermagem do Porto (ESEP). The recruitment process was meant to start in October, but due to the COVID-19 pandemic, it was suspended. We expect to restart the study by the beginning of the third quarter of 2021.

**Conclusions:**

The findings of this study protocol will contribute to the design and development of future robust studies for technological tests in a clinical context.

**Trial Registration:**

ISRCTN 15895163; https://www.isrctn.com/ISRCTN15895163

**International Registered Report Identifier (IRRID):**

PRR1-10.2196/25781

## Introduction

According to the United Nations, it is estimated that by 2050, the number of people aged 80 years and older will have increased by 3 times [1]. Increased longevity is often accompanied by structural and functional changes occurring throughout an individual’s lifespan and can be aggravated by certain behaviors, lifestyles, and environmental exposures, among other reasons. Some of the outcomes associated with these changes are loss of muscle strength, decreased balance control, and mobility impairments, which are risk factors strongly associated with the occurrence of falls in this population [2].

Globally, falls are highly prevalent in the elderly [3-5]. Although some asymmetries exist, it has been reported that about one-third of home-dwelling adults fall at least once a year, and of those who fall, two-thirds will experience another fall in the following year [4,6]. In this segment of the population, falls are complex events with multiple, dynamic interacting factors likely to lead to an increase in the frequency of such incidents with major implications on the quality of life [2]. The consequences of a fall can be extremely serious for the elderly and their families, as these falls can initiate or accelerate a vicious cycle of losses that can ultimately result in functional dependence leading to institutionalization [7,8]. Scientific research has identified several risk factors and has confirmed that the risk of falling increases with the number of existing risk factors [9]. Changes in gait and balance [2,9,10], decreased muscle strength [10], sensory deficits [2,10], functional decline [10], cognitive decline [2,9], musculoskeletal disease [4], neurological disease [2], endocrine or metabolic diseases [10], depression or depressive symptoms [9], urinary incontinence [10], the fear of falling [7,10], polymedication [2,9], and a history of a previous fall [2,4,9,10] are factors strongly associated with falls in the elderly. In a community context, research shows that the multifactorial web associated with these events also integrates risk factors present in household environments where the elderly perform their activities of daily living (ADLs) [11]; the synergistic and dynamic interaction of these risk factors with intrinsic factors increases the risk of falling.

Primary health care professionals should consider the existing evidence to develop interventions targeted at the elderly and their families [12] to identify and mitigate the environmental risks, and to promote safe behaviours. This intervention must include several programs effective for fall prevention, particularly when coupled with other approaches [9,11-13]. Changes in gait and balance are major factors associated with falls in the elderly, and rapid tests are recommended for the assessment of changes in gait and balance, such as the 30-second chair stand test (CST) [14], 4 stage balance test (4 SBT) [15], and timed-up and go test (TUGT) [9,16].

Despite the continued undervaluation of the importance of knowledge on fall prevention among the elderly population [11] by primary care health professionals, several fall prevention programs (single or multifaceted) have demonstrated a significant reduction in the risk of falls, number of falls, and fall-related injuries [6,9,13,17]. In fall prevention programs, an exercise component, either as a single intervention or integrated into multifaceted interventions, has proven effective in preventing these events and reducing associated injuries [6,9]. The Otago Exercise Program (OEP) developed at the University of Otago Medical School is an exercise program for fall prevention used in a community context internationally. The efficacy of the OEP has been attested in 4 randomized studies and 1 controlled multicenter study [6]. The focus of the OEP is to improve strength and balance with a simple, affordable, home-implemented, 12-month solution, which is monitored by a health professional through telephone interviews and home visits. The OEP was designed to be carried out autonomously by people in their homes, supported by a paper-based manual, after training with a physiotherapist for 4 individual sessions. Subsequently, other professional groups such as nurses have successfully administered the program [17,18].

Recent studies have strived to integrate technology into physical exercise programs that were revealed as effective for adherence and overcoming barriers to exercise, as well as for improvements in health and independence [19]. Currently, several technological solutions address specific aspects of the fall cycle; however, the majority of these do not address fall detection, fall risk assessment, and fall prevention simultaneously. The FRADE (Pervasive Platform for Fall Risk Assessment, Detection and Prevention) project seeks to develop a common technological platform that allows the integration of all these components. This platform consists of the use of sensors that will collect data and monitor the gait of the elderly. This platform can also send caregivers alerts and text messages in the event of a fall. The tablet application provides a set of fall prevention exercises based on the Otago Program; along with a wearable sensor, it allows for the monitoring of user performance and evaluation of progress. This study aims to assess the impact of an exercise program supported by a technological platform on functional variables associated with risk of falling and assess the ease of use of this technology by the elderly.

## Methods

### Study Design

A quasi-experimental study (before and after; single group) was carried out. Two research centres were involved in the project, the Nursing School of Porto (ESEP) and the Fraunhofer Portugal Assistive Information and Communication Solutions (AICOS).

### Recruitment and Sample

Participants aged 65 years or older who were living at home in the district of Porto, Portugal were recruited based on the following criteria.

The inclusion criteria are able to walk independently; cognitive impairment, as assessed by the Portuguese version of the Mini-Mental State Examination (cut-off points of 22 for 0-2 years of schooling, 24 for 3-6 years of schooling, and 27 for ≥7 years of schooling); no severe visual or hearing impairment; and willingness to participate in a physical exercise program with technological support.

The exclusion criteria are a self-reported chronic or acute illness for which exercise is contraindicated, history of hip or knee surgery or lower limb fracture in the last 12 months, current or previous participation in physical exercise programs in the last 6 months, and participation in another research study involving fall prevention programs.

After approval by the ESEP ethics committee, the recruitment of participants will start through the Living COLABORAR Network. This network is part of a Fraunhofer Portugal research center, and it participates in an organization of institutions that give social, health, and leisure support and promote the well-being of their clients, especially the elderly. Potential participants will be contacted by Fraunhofer researchers who will present the study objectives and collect information from people interested in participating in the study.

Subsequently, those willing to participate will be scheduled for an evaluation conducted at home by a rehabilitation nurse in 2 phases. The first phase involves screening potential participants based on the inclusion and exclusion criteria. Consequently, the questionnaire prepared for this study will be completed. These participants will spend a part of their days in the community center, where they will become familiar with the tablets.

The convenience sample will include at least 30 participants.

### Setting

This study will be deployed in the city of Porto, within the area of Bonfim at the council in which the care center is located. The population of Bonfim is about 24,265 people, of which 27.1% are elders (>65 years old), according to the 2011 census. This day center is located in an area of the city with an aging population, in which people aged 65 years and over represent 36.3% of the population and young people represent 18.2% of the population, with a total dependency index of 54.9%.

The elderly institution has 2 modalities: long-term hospitalization (for very dependent people) and a community center. The participants for this intervention will only be enrolled from the elderly who attend the activities in the community center but live in their own homes. The intervention will take place in the houses of the participants.

### Informed Consent

Participation in the study is voluntary, and participants are free to withdraw from the study at any time. All participants will provide written informed consent before data collection begins in the first home visit. The benefits, risks, and guarantee of confidentiality during data collection and information security for the participants are all explained in the consent form, in addition to the detailed explanation provided by the rehabilitation nurses.

### Intervention

The physical exercise program to be implemented is based on the OEP, including exercises aimed at improving balance, gait, and muscular strength in the lower limbs. The program is carried out by the elderly independently in their homes, supported by a paper-based manual, after in-person training with a health professional. In addition to the manual, the participants will have a common technological platform consisting of an Android tablet and a wearable sensor, which will enable them to access the OEP through interfaces with interactive feedback while exercising. This technological platform also allows interactive monitoring of the 5 strength exercises and 3 balance exercises (such as knee flexion, unipedal balance, and sit-and-stand).

In the first session at the participants’ homes, the rehabilitation nurse will train the participants to perform the exercises. Participants will be encouraged to carry out this program at least 3 times a week for 8 consecutive weeks. The rehabilitation nurse will provide participants with an in-person or telephone follow-up during these weeks.

The rehabilitation nurse will also instruct the elderly to wear comfortable clothes and shoes and inform the nurse if they experience joint or muscle pain. If such an event occurs, it is advisable to suspend the exercise.

Through the technological platform, the rehabilitation nurse will prescribe the OEP exercise plan for each participant, defining the exercises to be performed and the appropriate level of each exercise along with the frequency.

Each exercise session will have 3 sequential phases: warm-up phase, main phase, and relaxation phase with stretching exercises, which is a return to the calm phase. The progression in the exercises will be guided by the rehabilitation nurse and will be adapted to the functional capacity of the elderly. Regarding the intensity of the exercises, these will be performed without weights.

**Figure 1:**
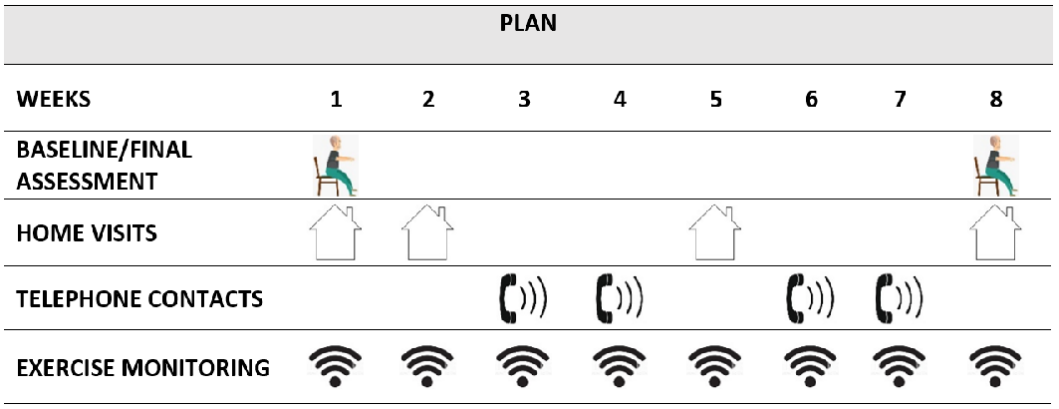
Intervention schedule.

The OEP exercises and phases are explained in Multimedia Appendix 1. Throughout the duration of the program, participants will be followed up in person and by telephone by the rehabilitation nurse according to the intervention schedule (Figure 1).

### Outcome Measures

A rehabilitation nurse with professional experience will assess the participant at the baseline visit and after 8 weeks using a questionnaire and functional test assessment. In addition to sociodemographic or clinical (self-reported) variables and environmental risk variables, the data collection tool integrates primary and secondary outcome variables.

The primary outcome is gait (TUGT; normal step) [16]. The secondary outcome variables are lower limb strength and muscle resistance (30-second CST) [14], the 4 SBT, frequency of falls, functional capacity (Lawton and Brody - Portuguese version) [20], Falls Efficacy Scale International (FES-I) Portuguese version [21], usability of the technology (System Usability Scale [SUS] Portuguese version) [22], and environmental risk variables (home fall prevention checklist [HFPC] for older adults) [23].

The change over time in the participants is measured by improvement in the functional test scores.

### Materials

**Figure 2:**
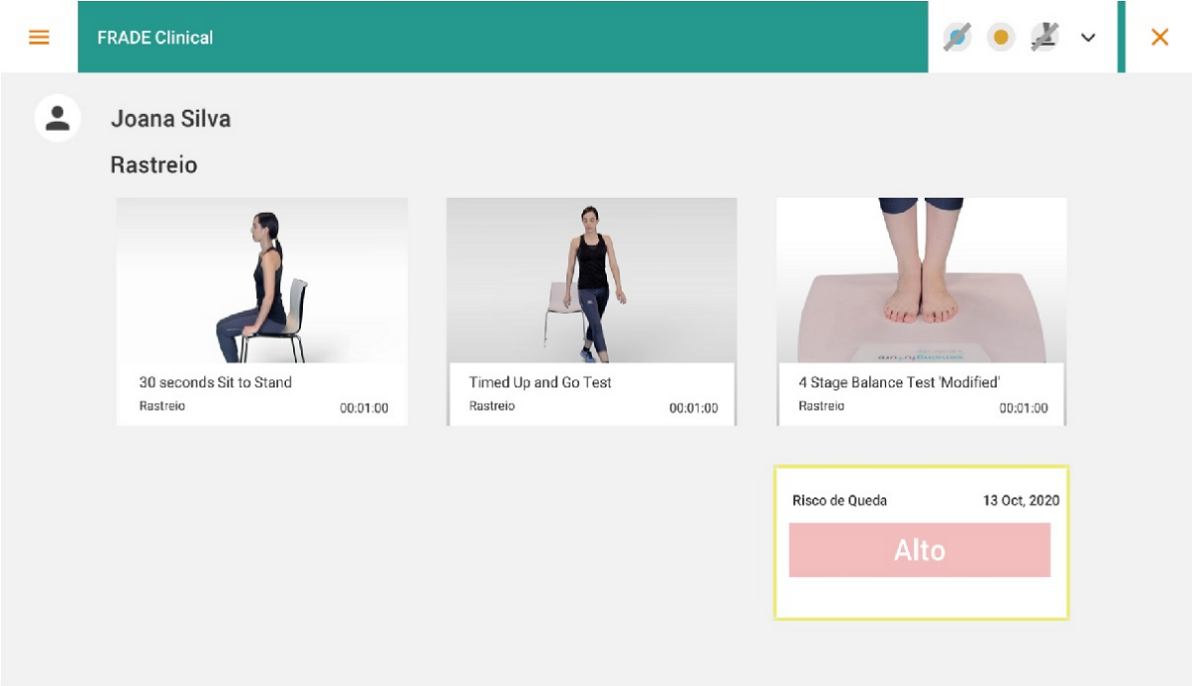
Fall risk screening tool.

The data collection instrument includes sociodemographic or clinical variables and functional evaluation variables. Lower limb strength and muscle resistance are the functional variables assessed through the 30-second CST, and mobility is evaluated with the TUGT (normal step). These functional tests, together with the 4 SBT, help evaluate the risk of a fall, which is also assessed with the fall risk screening tool using its inertial sensors to obtain information on the movements performed by the participant and related characterization. The risk of fall is then determined from parameters calculated after processing the signal from the inertial sensors during the execution of functional tests, such as walking, sitting, and standing (Figure 2).

**Figure 3:**
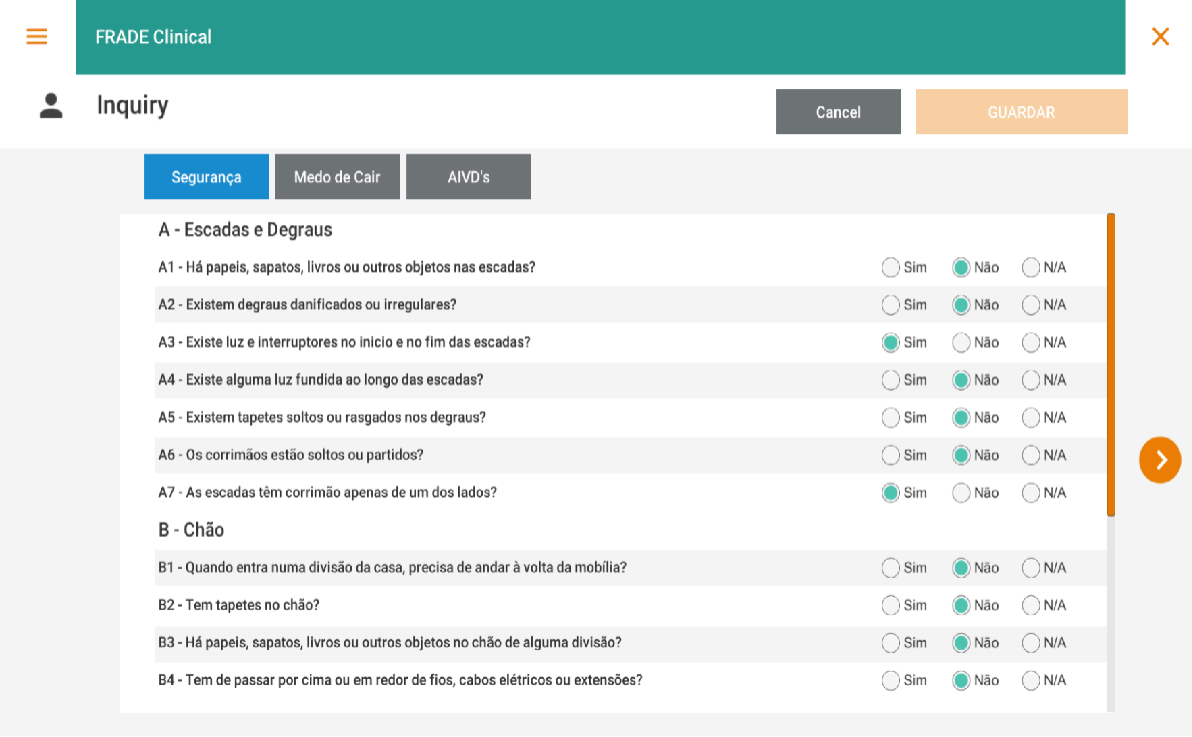
Data collection instrument.

The participants’ functional capacity for instrumental activities of daily living (IADL) is assessed using the Lawton and Brody tool, FES-I, SUS, and HFPC (Figure 3).

### Tests

The following tests will be performed to assess the outcome variables of the study.

#### Lower Limb Strength

The 30-second CST is a quick test that does not require a dynamometer, training, or special equipment. It is used to evaluate the strength of the lower limbs by counting the number of times the individual stands and sits in 30 seconds [14,24]. Therefore, the performance in the 30-second stand-and-sit test is used as a measure of the strength and muscle resistance of the lower limbs, specifically of the extensor muscles of the knee [25,26].

#### Mobility

The TUGT (normal step) measures, in seconds, the time an individual takes to stand up, walk a distance of 3 meters, return, and sit down in the same chair. Individuals who complete the test in <20 seconds are reportedly independent in manual handling transfer, and individuals who complete the test in >30 seconds tend to be dependent for this task [27]. It has also been reported that the time spent on the TUGT performance is related to scores on the Berg Balance Scale (*r*=–0.72), walking speed (*r*=–0.55) and the Barthel ADL index score (*r*=–0.51). The TUGT is a useful and practical measure for assessing mobility in the frail elderly and is easy and quick to apply without the need for special equipment or training.

#### Static Balance

The 4 SBT is used to track impairments in the static balance of the elderly. Several authors have reported that the test has excellent test-retest (*r*=0.97) and interevaluator reliabilities (kappa=0.92) [28,29]. According to the guidelines issued by the Centers for Disease Control and Prevention, the test must first be demonstrated to the participants by a nurse or physiotherapist, allowing the participants to perform a trial test to ensure correct performance. Participants are instructed to perform the four 10-second stages of the test sequentially. If the participant manages to perform 1 stage in 10 seconds, without moving the feet, losing balance, or needing support, the participant can move on to the next stage. If the participant fails this test, it is terminated [30]. The success of fall prevention programs is measured by comparing the positions achieved in 10 seconds in the pre- and postprogram evaluations [17].

Participants are instructed to be in an orthostatic position and perform the 4 different feet positions sequentially: position 1 is a side-by-side stance; position 2 is a semitandem stance (preferred foot forward with the instep foot touching the hallux of the other foot); position 3 is a tandem stance (one foot in front of the other, the heel of one foot touching the toes of the other); position 4 is a one-legged stance (using the preferred leg for support).

The final score will be the number of positions that are completed without loss of balance. Participants who cannot maintain position 3 for 10 seconds have a high risk of falling [30].

#### Functional Ability

The Lawton and Brody scale assesses the level of independence in performing IADLs, which include daily tasks such as using the telephone, shopping, cooking, housekeeping, washing clothes, using transport, preparing medication, and handling finances. It is an easy-to-administer tool that can be used in community and hospital settings [31-33].

In this study, the Portuguese version [20] of the Lawton and Brody scale is used, including the same items as in the original version, but with a polychotomic score (0, 1, 2, 3, or 4) instead of the original dichotomic version (0 and 1). This allows for a better description of an individual’s ability to perform the tasks, with a different score for each response option. The total score of the scale can be between 0 and 23, with a lower score corresponding to worse performance. In a validation study for this scale conducted with a sample of elderly people living in urban and rural settings, good metric qualities were shown when applied in a community setting, with a Cronbach alpha indicative of good reliability (0.94) and correlations between the scale items and the total scale between *r*=0.77 and *r*=0.86. In a study on the convergent validity of this scale, a strongly positive and statistically significant correlation with the Barthel Index was observed.

#### Fear of Falling

The FES-I evaluates the fear of falling. The elderly may have problems expressing their fear of falling. The assessment of this fear is highly relevant because it is associated with adverse effects on elderly mobility and quality of life [34-36].

One of the instruments used to evaluate the fear of falling is the FES-I [37] developed by ProFaNE (Prevention of Falls Network Europe Group) based on the original version of the FES developed in 1990 [38]. The FES-I incorporates 16 activities, including some daily activities slightly more complex than those in the original version, with others more targeted at the social life of elderly to strengthen some of the weak points mentioned in the literature about the original version. Its adaptation to different languages and cultural contexts (following the protocol recommended by the ProFaNE group) has allowed the scale to be widely applied, along with comparison of the results in different populations and contexts.

Because this scale provides an understandable measure of the fear of falling in the elderly and has excellent metric properties, the FES-I was chosen to measure the fear of falling in our study [39-41].

The Portuguese version of the FES-I [21] showed excellent internal consistency (Cronbach alpha=0.978) and test-retest reliability (ICC_2.1_=0.999). Concurrent validity assessed using the activities-specific balance confidence (ABC) scale showed results indicative of good concurrent validity (*r_s_*=–0.85; *P*<.001). Based on these results, the authors consider the Portuguese version of FES-I a reliable and valid measure to assess the fear of falling among the Portuguese community-dwelling elderly population.

#### Technology Usability

Technology usability will be analyzed using the SUS. The original author of the test describes it as a quick tool to assess the usability of a particular product or service [42]. According to some authors, this test has several features that provide good assessment of the overall usability, such as the flexibility to evaluate interface technologies, interactive voice response systems, and hardware used in more traditional computer interfaces and websites. Ease and speed of use, both by participants and system administrators; ease of operation of scoring; and free access are characteristic of this test [43].

The original SUS consists of 10 statements that are scored on a 5-point Likert scale (Strongly disagree – Strongly agree). The final score can vary from 0 to 100 points, with higher scores indicating better usability [42]. In this scale, an excellent score is above 90; a good score is above 80, and an acceptable score is above 70; scores below 70 indicate usability problems [44]. In 2015, a group of Portuguese researchers [22] began the translation and cultural adaptation of this scale along with the subsequent evaluation of its metric qualities. The same authors stated that the SUS could be used to distinguish between usable and nonusable applications.

### Procedures

#### Developing the Intervention

Meetings will be held between main investigators and rehabilitation nurses to present the study protocol. Subsequently, meetings will be held between rehabilitation nurses and the engineers who developed the applications. The first meeting will aim to present the clinical application (FallSensing Clinical App) to nurses. At the second meeting, the application to be used by the elderly in the home context (FallSensing Home App) will be presented, and a final meeting will be held to present the web platform. A screening test session will also be performed using all the tools.

The rehabilitation nurses will be provided with a checklist to standardize the procedures during the intervention phase, both for in-person and telephone follow-ups.

#### Ensuring the Safety of the Intervention

During the exercise program, the participants will be instructed to follow safety measures, such as performing the exercises in a large space without obstacles, along with wearing loose and comfortable sports clothes.

The nurse will evaluate the best ergonomic place in the house to perform the exercises.

In March 2020, the World Health Organization declared COVID-19 an international pandemic. Following this event, several important measures must be adopted to contain the spread of the disease, namely the use of a mask, social distancing, and hand hygiene.

Strict measures will be added to prevent the spread of COVID-19 in the nurses’ procedures when dealing with home-dwelling elderly, specifically during the execution of the physical exercise program. This means wearing a surgical mask, protective gown, and gloves; the personal protective equipment will be discarded after each contact.

#### Technological Platform

The technological solutions to be used to support the physical exercise program consist of 3 applications.

The FallSensing Clinical App requires the use of a Windows computer, 2 wearable devices with inertial measurement units, and a pressure platform to obtain information about the movement and balance of the user while performing the CST, TUGT, and 4 SBT. The application also allows the creation of a personal profile for each participant, in which the participants’ answers to questionnaires for the assessment of several risk factors for falls, such as the IADL or FES-I home hazards questionnaires, can be saved. Additionally, the application will enable the prescription of OEP exercises and scheduling for each participant through a dedicated exercise prescription interface. This exercise prescription will be sent automatically to the FallSensing Home application.

**Figure 4:**
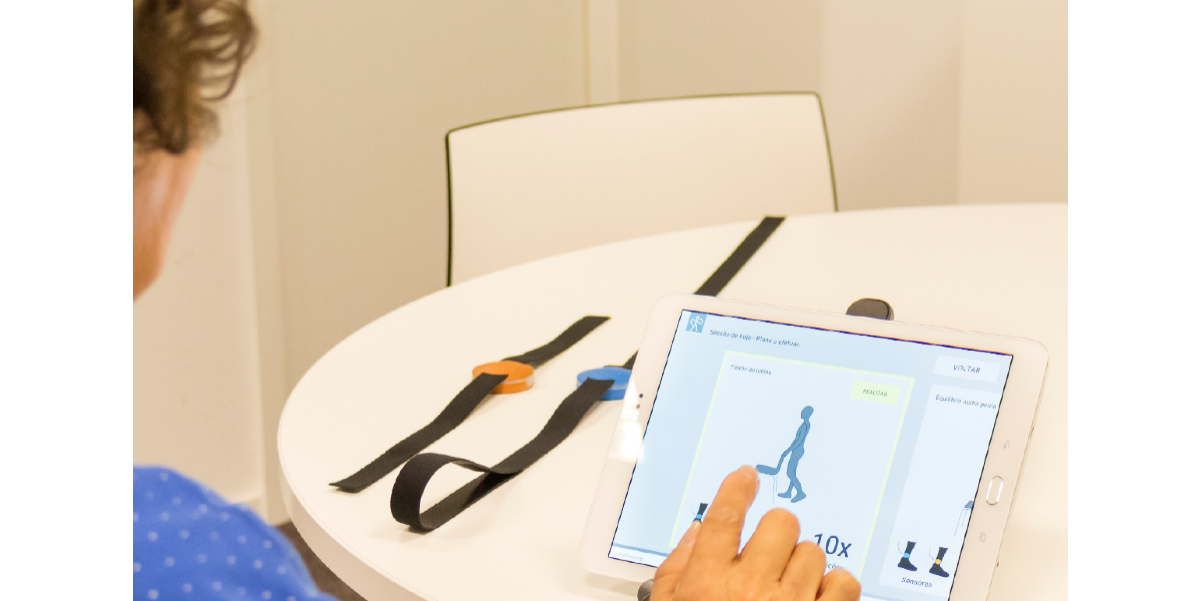
FallSensing Home App.

**Figure 5:**
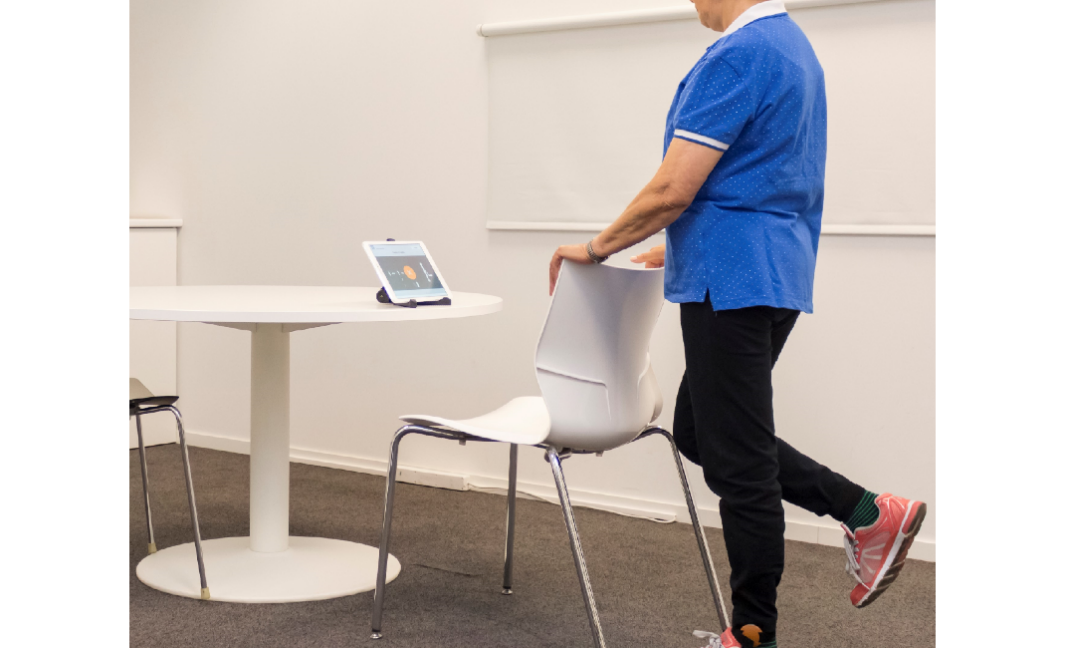
Demonstration of exercises through the FallSensing Home App.

The interactive FallSensing Home App, based on the OEP, aims to improve physical functionality. The application features 8 exercises from the program that are static, easy, and well accepted, with interactive feedback for the execution of the movement. The application can also motivate participants who perform the exercises at home. The application requires an Android tablet, support for the tablet, 2 wearable inertial devices, and the respective chargers. The users will be guided through a weekly exercise plan, which they will be able to select through the tablet interface where the instructions for executing each exercise will be presented, as well as an interface with visual feedback during the execution of each exercise (Figures 4 and 5).

The wearable fall detection device contains an automatic fall detection algorithm and offers features such as the sending of alarms through the wearable device, which can be placed discreetly on the belt, in the pocket, or on the chest of the user. If a fall event is detected, a notification will be sent to a backend that in turn sends a text message and email to a set of predefined emergency contacts. Communication between the device and the backend is via Narrow Band-IOT (Internet-of-Things). The device also triggers an audible alarm to attract the attention of people nearby, featuring an emergency button that allows the cancellation of false alarms. The device works independently of a smartphone or other resource and only needs to be charged via an induction charger, which will be supplied with the device.

**Figure 6:**
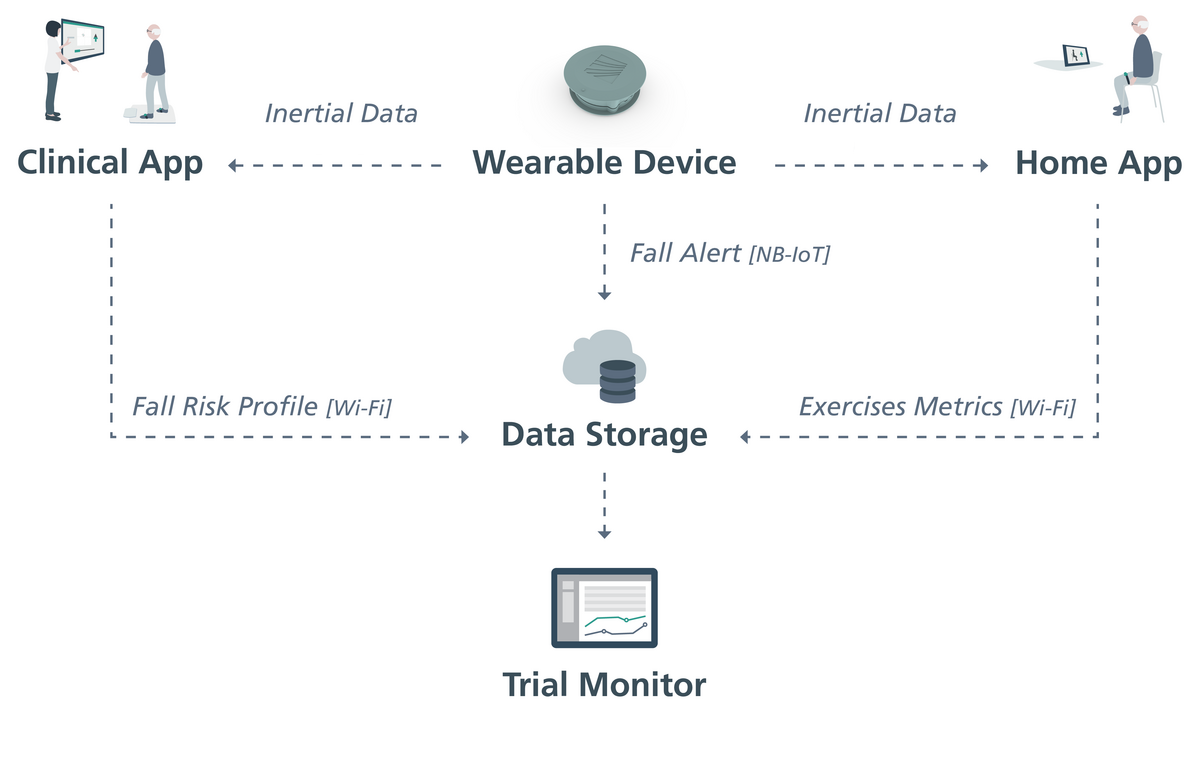
Process followed by the technological platform. NB-IoT: Narrow Band-Internet of Things.

Figure 6 facilitates a better understanding of the process of the technological platform.

### Statistical Analysis

Data processing will be carried out with SPSS software (IBM Corp, Armonk, NY). In the first approach, the data will be analyzed using descriptive statistics in order to classify the group of participants based on their sociodemographic, clinical, functional, and body processes data, using measures of frequency, central tendency, and dispersion. For inferential statistics, nonparametric statistics will be used, considering the sample size and sample type (nonrandom), with statistical significance set at *P*<.05. The prospective differences in the primary and secondary outcome variables, based on the 2 repeated measurements, will be tested using nonparametric tests equivalent to the *t* test for paired samples (Wilcoxon or McNemar procedure for quantitative and qualitative variables, respectively).

## Results

The recruitment process was meant to start in October 2020; however, due to the COVID-19 pandemic, it was suspended. We expect to restart the study by the beginning of the third quarter of 2021. The study results are expected to be published after this. This research will be carried out by a partnership between the ESEP and Fraunhofer Portugal. From this collaboration emerges a multidisciplinary team of nurse researchers, rehabilitation nurses, and engineers. This research seeks to obtain health gains for the elderly population.

This study was approved by the Ethics Committee of ESEP on October 10, 2020 (António Bernardino de Almeida, 4200-072 PORTO - Portugal; ref annex 2 meeting minutes no. 6/2020).

## Discussion

The study will provide an objective measurement of fall risk factors and movement-based metrics obtained during the OEP exercises, such as strength of the lower limbs, mobility, and balance impairment

A quasi-experimental study protocol will be developed, focusing on the OEP, involving home-dwelling elderly, and using a common technological platform, with follow-up by a rehabilitation nurse 3 times a week over 8 weeks.

Throughout the intervention, the follow-up of participants through regular home visits, coupled with telephone contact, will contribute to better monitoring of the evolution of the condition of the participant and will increase their confidence and security, as a determinant for the recognition of the role of the nurses.

The technological platform will allow the centralization of all relevant variables in a unified and secured database, which will be accessible through a web portal and made available for the nurses supervising the study. In addition to the variables retrieved by the clinical application (eg, personal profile, medical conditions, medication, answers to the questionnaires, and scores of the 3 functional tests, the home application will allow the measurement of the range of motion along with the number of repetitions and durations of ascending and descending movements for the 8 exercises of the OEP, namely, knee flexion, knee extension, hip abduction, knee bending, toe raises, calf raises, sit-to-stand, and one-leg standing exercises. Previous studies have set a background for the technological solutions used in this study [45,46].

This study has some limitations, namely, the use of a nonrandom sample and the absence of a control group. Additionally, the recruitment process was hindered by the COVID-19 outbreak, even though all the National Health Service guidelines were followed. Additionally, the funding agency provided a relatively short time for the development of the study.

As for the technological aspect, we consider that the technological literacy of each participant may affect an individual’s use of the platform and that some limitations are likely to arise during the 8-week intervention program since some of the sessions will be performed by the elderly alone at home. To attempt to overcome these limitations, frequent contact between the nurses and the elderly will be established along with remote guidance whenever deemed necessary.

In conclusion, the findings of this study protocol will contribute to the design and development of future robust studies for technological tests in a clinical context.

## Appendix

Multimedia Appendix 1 Otago Exercise Program exercises and phases.

## Abbreviations

ABC: activities-specific balance confidence
ADL: activities of daily living
AICOS: Center for Assistive Information and Communication Solutions
CST: chair stand test
ESEP: Escola Superior de Enfermagem do Porto
FES-I: Falls Efficacy Scale - International
FRADE: Pervasive Platform for Fall Risk Assessment, Detection and Prevention
HFPC: home fall prevention checklist
IADL: Instrumental Activities of Daily Living
OEP: Otago Exercise Program
ProFaNE: Prevention of Falls Network Europe Group
SBT: stage balance test
SUS: System Usability Scale
TUGT: timed-up and go test
