# Supplementary material for: A Technological-Based Platform for Risk Assessment, Detection, and Prevention of Falls Among Home-Dwelling Older Adults: Protocol for a Quasi-Experimental Study
Source: JMIR Res Protoc. 2021 Aug 12;10(8):e25781. doi: 10.2196/25781 (PMC8391727; doi:10.2196/25781)
Supplement: Multimedia Appendix 1 [file resprot_v10i8e25781_app1.doc]

**THE OTAGO EXERCISE PROGRAM MANUAL** ^1,2^

In the exercise session, the rehabilitation nurse will perform a demonstration and start with the warm-up exercises recommended by the OEP (WARM-UP PHASE), followed by lower limbs muscle strength exercises, improve balance and stability (MAIN PHASE) and finally a relaxation phase with stretching exercises (RETURN TO CALM PHASE).

**Warm-up** (6 stretching exercises)

1. **Walking**: Stand tall and start with a warm-up marching on the spot (use a chair for support if needed)

March for 1 to 2 minutes

1. **Neck rotation** (same as moving head from side to side):

Stand tall, feet waist-width apart and arms relaxed (use a chair for support if needed) Slowly turn your head to the left and then to the right

Keep shoulders straight

Move only your head

Repeat the exercise up to 5 times

1. **Neck extension** (same as moving head backwards):

Stand tall with feet waist-width apart (use a chair for support if needed)

Slowly guide your head straight back using 2 fingers (index and middle finger) placed on the chin until you feel the back of the neck stretching

Repeat the exercise up to 5 times

1. **Back extension**

Stand tall with feet shoulder-width apart and place both hands on your bottom.

Gently arch your back

Avoid looking at the ceiling or stretching your knees

Repeat the exercise up to 5 times

1. **Trunk movements**

Stand tall with feet waist-width apart (use a chair for support if needed)

Place your hands on your hips

Turn your trunk (head and shoulders) as far as you comfortably can to the right and then to the left

Do not move your hips

Repeat the exercise up to 5 times

1. **Plantar flexion and ankle dorsiflexion** (same as ankle relaxation):

Sit on a chair with your back against the back of the chair

Slowly lift one leg off the floor to a comfortable position

Pull the foot towards you, then point the foot down

Repeat the exercise up to 5 times

Repeat with the other leg

**MAIN PHASE** (5 strength exercises)

1. **Knee movements (same as thigh strengthening):**

Sit on a chair with your back against the back of the chair

Place the elastic band under one foot and hold it with both hands at knee level

Gently perform knee extension

Slowly lower your leg

Repeat the exercise up to 10 times with one leg and then with the other

Count to 3 when lifting your leg and slowly count to 5 when lowering it

1. **Knee flexion/extension** (same as posterior thigh strengthening):

Stand tall with feet waist-width apart and knees relaxed (use a chair for support if needed)

Slowly bend your knee and lift your heel towards bottom

Keep your knees close together

Slowly lower your foot

Repeat the exercise up to 10 times with one leg and then with the other

Count to 3 when lifting your leg and slowly count to 5 when lowering it

1. **Thigh femoral abduction** (same as hip strengthening):

Stand tall with feet waist-width apart and use a chair for support

Slowly lift one leg to the side, toe pointed forward

Then slowly lower that leg Repeat the exercise up to 10 times with one leg and then with the other

Count to 3 when lifting your leg and slowly count to 5 when lowering it

1. **Plantar ankle flexors** (same as calf strengthening):

Stand tall with feet waist-width apart, use a chair for support

Slowly lift heels, stand on toes taking your weight to your toes

Then slowly lower your heels to the floor

Repeat the exercise up to 10 times

Count to 3 when lifting heels and slowly count to 5 when lowering them, for each movement

1. **Posterior ankle flexors**: Stand tall with feet waist-width apart, use a chair for support

Slowly raise your toes, taking your weight to your heels Then slowly lower your toes Repeat the exercise up to 10 times Count to 3 when lifting toes and slowly count to 5 when lowering them, for each movement

**MAIN PHASE** (12 balance exercises)

1. **Knee flexion** (with or without support) (same as bending knees):

Stand tall, hold a chair for support, feet waist-width apart and point feet forward Slowly bend your knees to a half squat

Return to normal stance and repeat up to 10 times

Over time, as strength and balance increase, progress to 3 sets of 10 repetitions

1. **Toe walking** (with or without support) (same as tiptoe walking):
2. Stand tall close to your support and hold it, feet waist-width apart

Lift your heels, stand on toes

Walk 10 steps forward on tiptoes

Place feet parallel and lower your heels

Pause, turn around and return to start

Walk on steady steps

Repeat the exercise up to 4 times

1. **Tandem static balance** (with or without support) (same as stand with one foot in front of the other):

Stand tall close to your support and hold it

Place one foot directly in front of the other, so your feet form a straight line

Look ahead and keep balance for 10 seconds

Rest in normal stance, feet parallel waist-width apart

Change foot position and repeat the exercise for about 10 seconds

1. **Tandem walking** (with or without support) (same as walking with one foot in front of the other):

Stand tall close to your support and hold it

Place one foot directly in front of the other, so your feet form a straight line.

Take 10 steps forward

Look ahead and keep balance

Rest in normal stance with feet parallel waist-width apart

Repeat the exercise in the other direction

1. **Unipodal balance** (with or without support) (same as one leg stand):

Stand tall close to your support and hold it

Stand in one leg and hold this position

Keep the leg off the floor

Look ahead and keep balance for 10 seconds

Change foot and repeat exercise for another 10 seconds

1. **Sideways walking** (with or without support):

Stand tall in front of your support and hold it.

Take 10 steps to one side, straight waist

Repeat in the other direction

1. **Heel walking** (with or without support):

Stand tall close to your support and hold it

Lift toes keeping knees relaxed and bottom aligned with body

Take 10 steps forward on heels

Look ahead and take steady steps

Pause, turn around, switch hands, walk in the other direction

1. **Sit to stand** (with or without support):

Grab a chair and sit straight

Place your feet on floor, knees bent

Lean forward slightly and stand up (place your hands on the chair for extra support, if needed)

Then slowly sit back (place your hands on the chair for extra support, if needed) Repeat the exercise 5 times.

Over time, as strength and balance increase, progress to more repetitions

Repeat the exercise 10 times

1. **Backwards walking** (with or without support):

Stand tall close to your support and hold it

Walk 10 steps backwards

Look ahead, take steady steps and keep back straight

Walk starting by placing toes on the floor followed by heels

Repeat in the other direction

1. **Backwards tandem walking** (with or without support):

Stand tall and look ahead

Place one foot directly behind the other, so your feet form a straight line

Take 10 steps backwards

Rest in normal stance with feet waist-width apart

Repeat the exercise in the other direction

1. **Walking and turning around (figure-eight movement**):

Circle normally in a figure-eight movement

Keep your back straight

Repeat the exercise up to 2 times

1. **Stair climbing:**

Hold on to the handrail without placing excessive pressure in your arms

Use the strongest leg to start, placing foot on the stair step

When you reach the top of the stairs, pause and rest

When walking down the stairs, use your weakest leg first

**RETURN TO CALM PHASE** (2 stretching exercises)

1. **Calf stretching**:

Sit on the edge of the chair, place your hands on the chair for extra support

Stretch one leg, keep heel on the floor

Pull your toes back until you feel the stretching of the calf

Hold this position for 10 to 15 seconds

Repeat the exercise with the other leg

1. **Isquiotibials stretching**: (posterior thigh area):

Sit on the edge of the chair and hold on to it

Stretch one leg, keep heel on the floor

Place both hands on the other leg and lean forward with back straight until you feel stretching of the posterior thigh area

Hold this position for 10 to 15 seconds

Repeat the exercise with the other leg

PROGRAMME EQUIPMENT:

- Firm chair
- Elastic band - for muscle strength exercises
- Sensors and ribbon tapes - the same sensor will be used for all applications, FallSensing Screening, FallSensing Home and Wearable Fall Detection
- Induction charger for the wearable sensor
- Tablet Android e placement support
- Hotspot Wi-Fi – to enable para connection of the FallSensing Home to the database
- Pressure platform (only for Screening)
- Measuring tape for the TUG (only for screening)
